# Hilfe, ich kann nicht rülpsen: Falldarstellung und Kurzübersicht zur retrograden krikopharyngealen Dysfunktion

**DOI:** 10.1007/s00106-023-01383-x

**Published:** 2023-10-20

**Authors:** Daniel Runggaldier, Ursula Colotto-Vith, Daniel Pohl, Jörg E. Bohlender

**Affiliations:** 1 Klinik für Otorhinolaryngologie, Head and Neck Surgery, Abt. für Phoniatrie und klinische Logopädie, Universitätsspital Zürich, Frauenklinikstrasse 24, 8091 Zürich, Schweiz; 2 Universität Zürich, Rämistrasse 71, 8006 Zürich, Schweiz; 3 Klinik für Gastroenterologie, Universitätsspital Zürich, Rämistrasse 100, 8091 Zürich, Schweiz

**Keywords:** Ruktus, Botulinum Toxin, Luftaufstoßen, M. cricopharyngeus, Hochauflösende Ösophagusmanometrie, Soziale Medien, Eructation, Belching, Cricopharyngeal muscle, High-resolution esophageal manometry, Social media

## Abstract

In dieser Kurzübersicht wird das erstmalig 2019 durch den Laryngologen Dr. Bastian beschriebene Syndrom der retrograden krikopharyngealen Dysfunktion (R-CPD) anhand eines klassischen Falls vorgestellt. Die Diagnose erfolgt in der Regel klinisch durch die klassischen Symptome wie beispielsweise die Unfähigkeit, Luft aufzustoßen, abdominales Blähgefühl und retrosternale gurgelnde Geräusche. Des Weiteren werden neue Möglichkeiten der Diagnosesicherung mittels hochauflösender Ösophagusmanometrie und die therapeutischen Möglichkeiten mittels Injektion von Botulinumtoxin in den M. cricopharyngeus beschrieben sowie die dazu publizierten Daten diskutiert.

## Abstract (en)

In this short communication, we discuss the recently described syndrome of retrograde cricopharyngeal dysfunction (R-CPD) with its first description in 2019 by the laryngologist Dr. Bastian. Diagnosis is generally based on typical clinical symptoms, e.g., the inability to belch, a bloated abdomen and retrosternal gurgling noises. We also describe high-resolution esophageal manometry as a new tool to further secure the diagnosis of R‑CPD, as well as therapeutic options such as botulinum toxin injections in the cricopharyngeal muscle or cricopharyngeal myotomy and the published data thereon.

## Anamnese

Die Vorstellung des 22-jährigen Patienten erfolgt aufgrund einer seit vielen Jahren bestehenden Unfähigkeit, Luft aufzustoßen (Ruktus). Der Patient beschreibt, auch bei kleineren Mahlzeiten unter einem ausgeprägten Gefühl von aufgeblähtem Bauch mit exzessiven Flatulenzen zu leiden. Verbunden ist diese Symptomatik mit gurgelnden Geräuschen hinter dem Brustbein. Die Aufnahme von kohlensäurehaltigen Flüssigkeiten (u. a. Mineralwasser, Sekt) wird vom Patienten stets als sehr unangenehm empfunden und meist bewusst vermieden. Insgesamt haben diese Beschwerden zu deutlichen Einschränkungen im sozialen Leben geführt. Abgesehen von einem gelegentlichen sauren Aufstoßen werden weitere Beschwerden im Hals-Nasen-Ohren-Bereich verneint. Es bestehen keine Dysphagie, keine Dyspnoe, keine Stimmveränderungen, keine Allergien oder Lebensmittelunverträglichkeiten. Keine Noxen. Keine Vorerkrankungen. Keine regelmäßige Medikation. Bezüglich der eindrücklich geschilderten Symptomatik erfolgten bereits im Vorfeld zahlreiche externe Abklärungen in der Allgemeinmedizin (körperliche Untersuchung, laborchemische Blutuntersuchung), HNO-Heilkunde und Gastroenterologie (u. a. mit Gastroskopie), welche allesamt unauffällig waren.

## Befund

Bei sehr gutem Allgemeinzustand zeigt sich ein unauffälliger Status im HNO-Bereich wie auch eine unauffällige transnasale Fiberendoskopie und Stroboskopie mit reizlosem Epi‑, Meso- und Hypopharynx. Der Larynx präsentiert sich reizlos, intakt mit beidseits symmetrisch beweglichen Stimmlippen und komplettem Glottisschluss bei Phonation. Keine vermehrten Speichelresiduen im Bereich der Sinus piriformes und Valleculae. Regelrechte Randkantenverschieblichkeit. Auditiv perzeptiv (Rauheit, Behauchtheit, Heiserkeit insgesamt). R0B0H0.

## Diagnostische Ergebnisse

**Figure 1:**
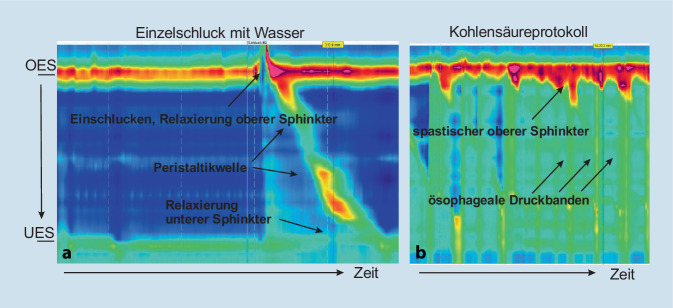

Bei anamnestisch hochgradigem Verdacht auf eine retrograde Dysfunktion des M. cricopharyngeus (R-CPD) und ansonsten unauffälligen Untersuchungsbefunden wurde zur weiteren Diagnosesicherung eine hochauflösende Ösophagusmanometrie (HRM) veranlasst (Abb. 1, Funktionslabor Universitätsspital Zürich, USZ, Flughafen). Hierbei zeigte sich ein regelrechter Ruhetonus des oberen und unteren Ösophagussphinkters sowie eine regelrechte Peristaltik in den Einzelschlucken mit Wasser (ohne Kohlensäure). Dabei konnte vor allem auch eine unauffällige anterograde Relaxation des oberen Ösophagussphinkters beim Einschlucken beobachtet werden (Abb. 1a). Nach raschem Trinken von kohlensäurehaltigem Mineralwasser (600 ml innerhalb von ca. 5 min, Kohlensäureprotokoll) berichtete der Patient von einem ausgeprägten Völlegefühl. Ein Aufstoßen des übermäßigen Gases war dabei nicht möglich. Manometrisch zeigten sich simultan zu dieser klinischen Symptomatik abdominale und ösophageale Druckerhöhungen mit einem spastischen Tonus des oberen Ösophagussphinkters mit fehlender Relaxation, sodass die Diagnose einer retrograden Funktionsstörung des M. cricopharyngeus gestellt werden konnte (Abb. 1b).

## Therapie und Verlauf

**Figure 2:**
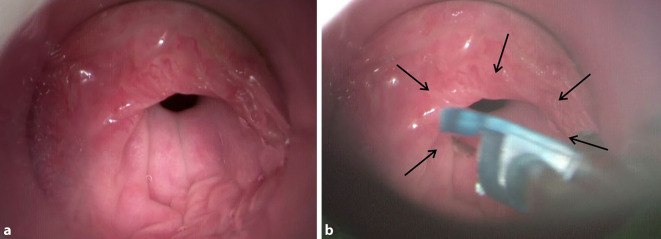

In Zusammenschau der Anamnese und der manometrischen Befunde wurde die Diagnose einer R‑CPD gestellt und eine Mikrolaryngoskopie mit Injektion von Botulinumtoxin in den M. cricopharyngeus durchgeführt. Intraoperativ zeigte sich dieser Muskel als prominenter zirkulärer Schleimhautwulst im proximalen Ösophagusbereich (Abb. 2a), mittels Butterfly-System wurden insgesamt 50 Einheiten Botulinumtoxin (Fa. AbbVie, Präparat Botox ®, 100 Allergan Einheiten pro Ampulle) injiziert (Abb. 2b).

**Figure 3:**
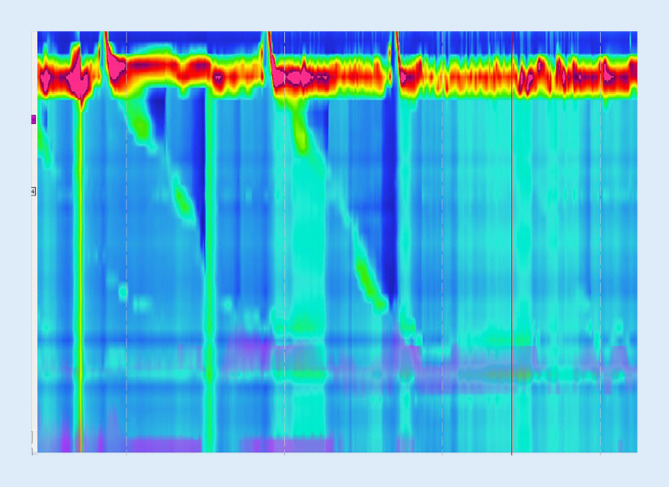

Postoperativ ergab sich im Verlauf eine langsame, aber kontinuierliche Besserung. Bereits einen Monat nach der Botulinumtoxininjektion konnte der Patient nach eigenen Angaben „nun richtig Luft aufstoßen“. Das postprandiale abdominale Blähgefühl, insbesondere auch nach Aufnahme von kohlensäurehaltigen Getränken, und die gurgelnden retrosternalen Geräusche waren ebenfalls regredient. Die postoperative Manometrie ca. 6 Wochen nach Botulinumtoxininjektion zeigte nun weniger ausgeprägte pathologische Veränderungen (Abb. 3).

## Diskussion

Die genannte Falldarstellung beschreibt die Diagnosestellung und Behandlung einer R‑CPD inklusive vorausgegangener apparativer manometrischer Diagnostik (HRM) mit Kohlensäureprotokoll.

Bei der R‑CPD handelt es sich um ein erstmalig 2019 durch Dr. Bastian (Voice Institute, Illinois, USA) beschriebenes Syndrom, das durch den in Tab. 1 beschriebenen Symptomkomplex charakterisiert ist [2]. Bereits vorgängig gab es zwar vereinzelt Falldarstellungen dieses Beschwerdekomplexes, bei dem retrograde Störungen des beim Ruktus involvierten Reflexbogens postuliert wurden. Allerdings wurden daraus noch keine therapeutischen Konsequenzen wie die der krikopharyngealen Botulinumtoxininjektionen durch die Autoren abgeleitet [4, 9]. Im für die Beschreibung des Syndroms relevanten Jahr 2015 wurde ein verzweifelter Patient jedoch mit klassischer R‑CPD-Symptomatik bei Bastian vorstellig und eine probatorische krikopharyngeale Botulinumtoxininjektion durchgeführt [2]. Der nun posttherapeutisch beschwerdefreie und zufriedene Patient veröffentlichte daraufhin ohne Kenntnis von Dr. Bastian auf der Internetplattform „Reddit.com“ seine Krankengeschichte, woraufhin zahlreiche weitere Patientinnen und Patienten mit R‑CPD auf Dr. Bastian aufmerksam und für eine Therapie selbstständig vorstellig wurden [2, 7].

**Table 1:** 

1	Unfähigkeit zum Luftaufstoßen	Inability to belch
2	Abdominales Völlegefühl vor allem nach dem Essen	Abdominal bloating esp. after eating
3	Gurgelnde Geräusche retrosternal als Folge des versuchten Luftaufstoßens	Gurgling noises from the chest to lower neck as esophagus is straining to eject air
4	Exzessive Flatulenz	Excessive flatulence
5	Einschränkung im sozialen Leben	Social inhibition
6	Schwierigkeiten beim Erbrechen (nicht bei allen Patienten beobachtet)	Difficulty vomiting (common but not universal)

Bislang ist die verfügbare Literatur und Datenlage der erstmalig 2019 beschriebenen R‑CPD dünn und die Bekanntheit dieser Störung in der medizinischen Fachwelt gering. An dieser Stelle ist zudem auch noch der immer relevantere Einfluss von Social Media zu unterstreichen: Neben Reddit.com gibt es mittlerweile zahlreiche Foren, Gruppen und Videos auf verschiedenen Plattformen wie TikTok, Facebook oder Youtube, die sich dem Thema R‑CPD widmen. Immer häufiger kommt es daher vor, dass sich Patientinnen und Patienten selbstständig mit der Verdachtsdiagnose einer R‑CPD bei einem Arzt vorstellen [2, 8].

Die Diagnosestellung erfolgt primär mittels Anamnese mit der in Tab. 1 beschriebenen Symptomatik und ansonsten unauffälligen Untersuchungsbefunden. Zur Diagnosesicherung ist in der Literatur zudem kürzlich auch die Möglichkeit einer HRM mit speziellem Kohlensäureprotokoll beschrieben worden [6]. Dabei nimmt der Patient innerhalb von kurzer Zeit ca. einen halben Liter kohlensäurehaltige Flüssigkeit zu sich. Bei Vorliegen einer R‑CPD können dann in der Manometrie klassischerweise abdominale und ösophageale Druckbanden bei gleichzeitig spastisch wirkendem oberen Ösophagussphinkter im Sinne einer retrograden Relaxationsstörung des M. cricopharyngeus beobachtet werden [6]. Dies deckt sich sehr gut mit unserem Fall, bei dem neben der charakteristischen Anamnese auch die R‑CPD typischen manometrischen Befunde demonstriert werden konnten (Abb. 1). Der Stellenwert dieser Diagnostik bleibt jedoch zum jetzigen Zeitpunkt aufgrund der begrenzten Daten unklar.

Es muss angenommen werden, dass eine R‑CPD mit einem erheblichen Leidensdruck für die betroffenen Patienten verbunden ist, welcher sich nach Botulinumtoxininjektion in den allermeisten Fällen ohne relevante Nebenwirklungen deutlich verbessern lässt [3, 5]. Interessant ist an den bereits publizierten Daten vor allem, dass der therapeutische Effekt zum Teil deutlich länger anzuhalten scheint, als sich aus der Wirkungsdauer des Botulinumtoxins im Gewebe ableiten lässt [3]. Gründe für dieses Phänomen sind derzeit noch nicht bekannt. Ebenfalls fehlen zum jetzigen Zeitpunkt Daten, die das Ansprechen auf mittlere oder längere Zeiträume von 5 oder mehr Jahren untersuchen. Gemäß Literatur kommt es jedoch bei einem kleineren Anteil der Patientinnen und Patienten mit einer R‑CPD nach einigen Monaten oder Jahren zu erneuten Beschwerden [3]. In diesem Fall oder bei fehlender Wirkung des Botulinumtoxins könnte als Alternative jedoch noch eine Myotomie des M. cricopharyngeus erwogen werden, wobei hierzu lediglich eine beschriebene Falldarstellung existiert und der Stellenwert dieser Option somit noch unklar ist [1].

## Fazit für die Praxis

- Bei Vorliegen des charakteristischen Symptomkomplexes mit einer Unfähigkeit zum Luftaufstoßen, abdominalem Völlegefühl vor allem nach Aufnahme von kohlensäurehaltigen Getränken, gurgelnden Geräuschen retrosternal, exzessiven Flatulenzen und Einschränkungen im sozialen Leben als Folge davon ist an eine R‑CPD zu denken.
- Eine Diagnosesicherung der R‑CPD kann mittels „Kohlensäureprotokoll“ im Rahmen einer hochauflösenden Manometrie untermauert werden.
- Eine Botulinumtoxininjektion in den M. cricopharyngeus kann in vielen Fällen zu einer langandauernden Beschwerdebesserung führen – länger als durch die Wirkungsdauer des Botulinumtoxins im Gewebe zu erwarten wäre.
- Diverse Social-Media-Plattformen haben maßgeblich zur Beschreibung und zum Bekanntwerden der R‑CPD beigetragen.
